# Intraoral scanner-based monitoring of tooth wear in young adults: 24-month results

**DOI:** 10.1007/s00784-023-04858-x

**Published:** 2023-01-10

**Authors:** Maximiliane Amelie Schlenz, Moritz Benedikt Schlenz, Bernd Wöstmann, Anna Sophia Glatt, Carolina Ganss

**Affiliations:** 1 Department of Prosthodontics, Dental Clinic of the Justus Liebig University Giessen, Schlangenzahl 14, 35392, Giessen, Germany; 2 Department of Conservative and Preventive Dentistry, Dental Clinic of the Justus Liebig University Giessen, Giessen, Germany; 3 Department of Operative Dentistry, Endodontics and Paediatric Dentistry, Section Cariology, Dental Clinic of the Philipps-University Marburg, Marburg, Germany

**Keywords:** Tooth wear, Erosion, Attrition, Young adults, Intraoral scanner, Monitoring

## Abstract

**Objectives:**

Tooth wear causes irreversible cumulated surface loss and already occurs at a young age. Therefore, the objective of this clinical prospective observational study was to monitor the occlusal surface of a mandibular first molar in young adults for a period of 24 months. Furthermore, potential aetiological factors obtained by a questionnaire were considered.

**Materials and methods:**

The study teeth (FDI #36 or #46) of 81 participants (mean age 22.8 ± 2.2 years) were scanned with the intraoral scanner (IOS, Trios 3, 3Shape) at the second follow-up (T2) after an observation period of 24 months. Standard-tessellation-language datasets were superimposed with baseline (T0) and T2 scans in 3D analysis software (GOM Inspect). The maximum vertical substance loss was measured between T0 and T2 at 6/7 areas (4/5 cusps and 2 ridges) of each study tooth and data compared to the already published data of the first follow-up (T1) after 12-month observation period. The morphology of tooth wear was classified into three groups: cupping (C), facet (F) and combined cupping-facet (CF). The analysis of aetiological factors, such as acid impacts, was based on a questionnaire filled out by participants at time points T0, T1 and T2. Non-parametric tests were used for statistical analysis (*p* < 0.05).

**Results:**

The buccal load-bearing cusps (mesiobuccal: median 15 μm, 95%CI 11/18 μm; mesiolingual 8 μm, 0/11 μm) were most affected by tooth wear. Loss values increased significantly at T2 compared to T1 for all areas, although significantly less than in the first 12 months (T0–T1). Areas that already exhibited F at T0 mostly displayed them also at T2 and only rarely developed further into C or CF. The only association between aetiological factors and loss values could be detected for sex as males had significantly higher loss values than females.

**Conclusions:**

Progression of tooth wear could be clearly shown with high interindividual variations in loss values among participants. This indicates the need for individual monitoring with IOS.

**Clinical relevance:**

IOSs show the potential for patient-specific monitoring to detect the progression of tooth wear. Thus, data of 24 months fills the gap of tooth wear data for young adults in literature. Further studies over a longer observation period are highly recommended to gain more information about the dynamic of tooth wear and aetiological factors.

## Introduction

Tooth wear is defined as surface loss of mineralized tooth substance due to physical or chemophysical processes [1]. The processes that are involved are attrition (loss of mineralized tooth substance caused by tooth-to-tooth contact), abrasion (physical loss of mineralized tooth substance caused by objects other than teeth) and erosion (chemical loss of mineralized tooth substance caused by the exposure to acids not derived from oral bacteria) [1]. Tooth wear is a cumulative physiological process; accordingly, the frequency and severity generally increase with age [2]. However, it can be regarded pathological when it is beyond the physiological level relative to the individual’s age and interferes with the self-perception of well-being [1].

The data on the prevalence of tooth wear of different aetiologies in younger age groups varies. Little data has been published on the prevalence of dental hard tissue loss due to attrition though it seems to affect almost all individuals [3] and is already common in adolescents [4]. Tooth wear due to abrasion, on the other hand, is a very heterogeneous phenomenon, which is why there is almost no prevalence data available. An exception are noncarious cervical lesions (NCCL), which are attributed at least in part to abrasion. A recent systematic review concluded that about 47% of adults are affected by NCCLs, with higher numbers in the older population [5]. In contrast, much more is published on the prevalence of erosive tooth wear and it appears that about 30% of adolescents and young adults are affected by this form of wear [6].

In addition to the prevalence, the incidence and progression of tooth wear is of interest, in particular in the younger age groups. Already at this age, an increase of wear by attrition [7, 8] but also triggered by erosion [9–11] can be observed. Especially in this period of life, it is important to consider such wear processes because of the long remaining lifespan of the dentition, to identify influencing factors and perhaps also to find risk indicators that point to pathological wear rates.

Today, it is possible to quantify and monitor the in vivo progression of tooth wear in the submillimeter range by superimposing serial digital models [12–14]. Compared to visual observation, whether clinical, on photographs or models, this offers the advantage of shorter observation times and objective quantitative measurement parameters that can then be linked to morphological aspects. Intraoral scanners (IOSs) in particular provide new perspectives, as these devices can be used to create digital models very quickly and without the need for conventional impression taking and cast fabrication. A recent study [15] used this method and showed that a group of 11–18-year-old adolescents lost an average of 0.013 ± 0.009 mm^3^ of dental hard tissue per square millimeter of tooth surface at the incisal edges of the upper anterior teeth and the occlusal surfaces of the mandibular first molars within 1 year. Latter are the most common teeth to exhibit these lesions and show the most pronounced progression rates [9, 10, 16–18]. Therefore, mandibular first molars have been proposed as marker teeth for erosive tooth wear in young subjects [16, 18].

**Fig. 1:**
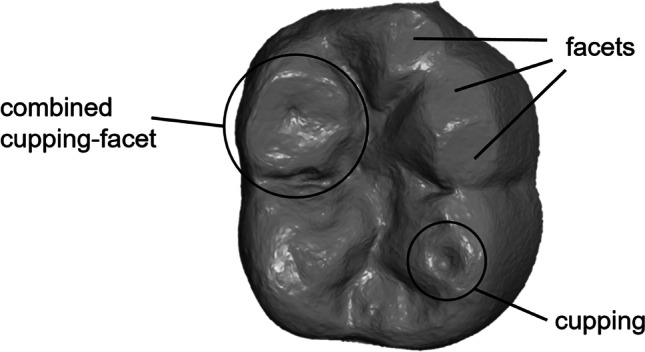
Example of the macroscopic morphologies: facets (F), cuppings (C) and combined cupping-facets (CF)

Previously published first data [19] from clinical wear measurements with an IOS of the occlusal surfaces of mandibular first molars in young adults after 12 months revealed that except for one tooth, all teeth examined showed signs of wear, with various macroscopic morphologies such as facets (F), cuppings (C) or combined cupping-facet (CF) (Fig. 1). On the one hand, this is part of physiological processes, but it can also make interventions necessary if the wear rate is high. Data showed that all such types of wear can be progressive, regardless of where they are located on the occlusal surface. Though median wear rates of less than 50 μm within 1 year may be considered physiological, the results of this study show a considerable range of values. The tooth area with the most wear and the greatest progression was the mesiobuccal one and subjects with losses in the 75% quartile presented values between 54 and 183 μm in this area. Because of the rather large range of wear rates, it would be plausible to assume that factors can be identified that are associated with higher loss rates. However, any nutrition-related or other influencing variables to explain this were not found, but the initial observation period of 12 months may be too short to detect such associations.

Therefore, a continuation of this study to collect further data on wear dynamics was sought. The question to be answered is to what extent tooth wear is progressing and whether its morphology is changing. Furthermore, the intention was to investigate whether the biological, nutrition-related and behavioural data, which could not associate with tooth wear during the first observation period, could be identified as influencing factors after a longer monitoring time.

The present clinical prospective observational study assessed the wear process on the occlusal surfaces of mandibular first molars in young adults. The primary aim was to collect qualitative and quantitative data on the wear processes in the natural dentition of young subjects. The secondary aim was to use a specifically designed questionnaire to explore the impact of various aetiological factors, as well as to relate the data regarding quantitative loss to the assumed aetiological factors obtained from the questionnaires. The study explores the use of IOSs as an innovative tool for the monitoring of tooth wear after a 24-month observation period.

## Participants, materials and methods

The study was conducted at the Department of Prosthodontics and the Department of Conservative and Preventive Dentistry of the Justus Liebig University Giessen (Germany) in compliance with the ethical guidelines of the Declaration of Helsinki. The clinical trial was approved by the local ethics committee of the Justus Liebig University Giessen (ref. no. 148/18) and registered in the German Clinical Trial Register (DRKS00021279). All participants were informed about the procedure and background before the start of the study. Furthermore, a declaration of consent has been signed. The observation period was between the end of 2018 and 2021.

The present study continues the monitoring of wear and follows up on the data from Schlenz et al. [19]. For this purpose, the data from T0 (baseline) and the data after 12 months of observation were taken over and are now extended by the 24-month data.

The study tooth was a first lower molar (FDI #36 or #46). Inclusion criteria were age between 18 and 25 years at baseline (T0), a lower first molar without caries or visible plaque and with occluding antagonists. Fillings should not exceed one-third of the occlusal surface. Exclusion criteria were severe general disease [20], current orthodontic treatment and maxillary crowns and bridges.

**Fig. 2:**
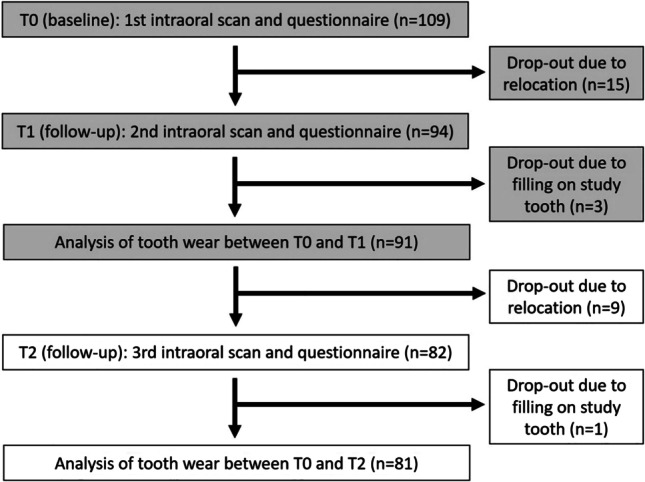
Flow scheme of the clinical study

Detailed information about the clinical examination, measurement of tooth wear and questionnaire is available in 12-month publication [19]. In brief, the study protocol is described below:

At the beginning of the present study (baseline T0), all study participants were aged between 18 and 25 years (21.0 ± 2.2 years, 62 % female; *n* = 109). The mean observation period for T0–T1 was 373 ± 19 days and for T0–T2 752 ± 51 days. Intraoral scans were captured with Trios 3 (3Shape, Copenhagen, Denmark) at T0, after 12-month follow-up (T1) and after 24-month follow-up (T2) by the same operator (M.A.S.) (Fig. 2). Before usage, the IOS was calibrated according to the manufacturer’s instructions, and the specified warm-up time was adhered [21]. During the scan procedure, which was taken as briefly as possible and strictly according to the recommended scan path, the light of the dental unit was switched off [22, 23]. Teeth were gently air-dried and dry tips (Microbrush International, Grafton, MA, USA) were used to absorb saliva of the parotid gland. All participants had to brush their teeth before scanning was performed. Only the respective study tooth (#36 or #46) with the adjacent teeth was scanned. For further analysis, scan data was saved in standard-tessellation-language (STL) format.

For the superimposition of IOS datasets and measurement of tooth wear, the STL datasets of T2 were imported into the 3D analysis software GOM Inspect (version V8 SR1, GOM GmbH, Braunschweig, Germany). First, scan data was cropped to the area of interest, which was defined as the occlusal surface above the anatomical equator of the study tooth defined as the equatorial line that separates the tooth in retentive and non-retentive areas. Then, datasets of T2 were superimposed to the pre-existing datasets of T0 of the previous study using the established best-fit alignment with the iterative closest point technique [24, 25]. In detail, this alignment method implicates first the initial best-fit alignment of the baseline dataset (T0) and the follow-up dataset after 24 months (T2). Following, the average overlay error will be determined and a further best-fit alignment using only those areas with a deviation less than the overlay error will be applied. This method was repeated until the overlay error did not decrease anymore. An average overlay error of ≤ 10 μm was achieved. Data of T0–T1 were already evaluated by same methodology [19].

**Fig. 3:**
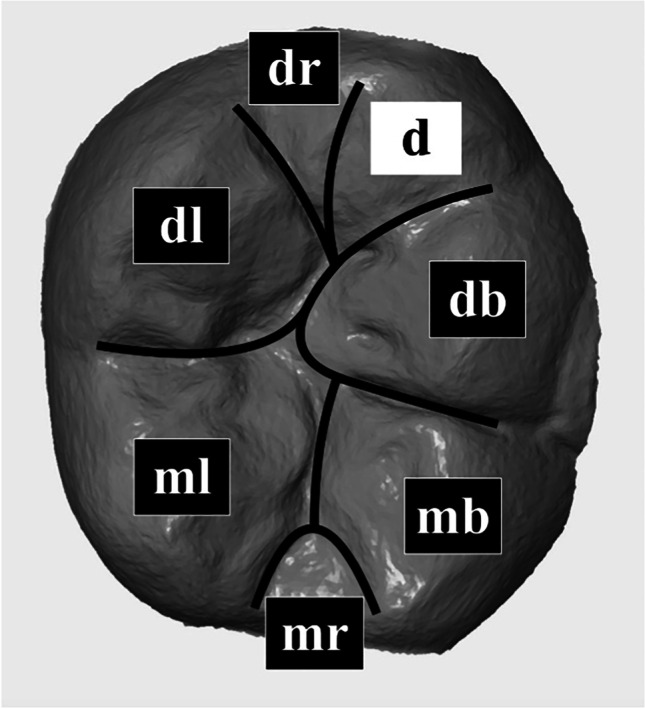
Distribution of the occlusal surface of each study tooth into seven areas: mesiobuccal (mb), distobuccal (db), mesiolingual (ml), distolingual (dl) and if existing distal (d) cusp as well as mesial (mr) and distal ridge (dr)

The measurement of maximum vertical tissue loss was conducted in six respectively seven areas of the occlusal surface (Fig. 3): mesiobuccal (mb), distobuccal (db), mesiolingual (ml), distolingual (dl) and, if existing, distal (d) cusps as well as mesial (mr) and distal marginal ridge (dr). In addition, the morphology of tooth wear was classified into three groups: cupping (C), facet (F) and combined cupping-facet (CF).

Additionally to the intraoral scanning procedure, all participants were asked to fill out a questionnaire, which was designed in cooperation with an experienced scientific nutritionist (A. J.) [19]. Potential associated factors to tooth wear such as nutrition behavior, the frequency of consumption of acidic food and beverages or chewing gum, the presence of heartburn and wearing a night guard were considered. With the question, of how much the participants generally liked acidic food and beverages, the authors tried to identify if there could be a certain taste preference. For the analysis, acid impacts per day were calculated according to the 12-month publication [19].

Statistical analyses were performed using IBM SPSS Statistics version 27 (IBM Germany GmbH, Ehningen, Germany). Loss values showed significant deviations from the Gaussian distribution (Kolmogorov-Smirnov test); therefore, non-parametric test procedures were generally used. Values are presented as median and 95% confidence intervals obtained by bootstrapping (method of sampling: simple, number of samples: 1000). The comparison of loss values at T1 and T2 was done using the Wilcoxon test and the wear rates from T0 to T1 and from T0 to T2 were correlated using Spearman’s rho. The morphology of wear at T0 and T2 is given descriptively with cross-tabulations. Kruskal-Wallis test and subsequent Mann-Whitney tests were used to investigate whether the loss values differed according to the wear morphology.

In order to identify factors that might influence wear, the focus was on a region exhibiting the highest tissue loss values in the present study as well as the greatest morphological variability already at baseline; this was the mesiobuccal cusp area, which was therefore defined as a region of particular interest. The relation of loss values to sex, as well as to the questionnaire items “wearing a night guard” and “consumption of chewing gum” was examined with the Mann-Whitney-*U* test and the relation of loss values to taste preferences with the Kruskal-Wallis test. Furthermore, the subjects were divided into those with C or CF and those with no morphological changes or F. The question of whether wearing a night guard, the consumption of chewing gum or taste preferences of these two groups differed was examined with cross-tabulations and Cramer’s phi, and whether the acid impacts of these two groups differed was examined with the Mann-Whitney-*U* test. Heartburn was reported rarely (*n* = 6); hence, it was not included in the statistical analysis. Loss values of the mesiobuccal area and acid impacts were correlated with Spearman’s rho. The level of significance was set to *p* < 0.05.

## Results

After 24 months (T2), data from 81 participants could be analysed, because out of the initially 109 participants, *n* = 24 dropped out due to relocation, and *n* = 4 were excluded because of restorative therapy on study tooth (Fig. 2). The mean age of participants was 22.8 ± 2.2 years (64 % female).

Table 1 shows the proportions of the wear morphologies in the different areas at T0 and their transformation after 24 months (T2). It was shown that the buccal load-bearing cusps, especially the mesiobuccal one, most frequently presented signs of wear, whereas the mesial and distal ridge were the least affected. As was already the case at T0, facets were most frequently found at T2, being present in 60% of all areas. In contrast, cupping and combined cupping-facet were found in only about 10% of the areas. The latter were almost exclusively located in the mesiobuccal area, which was affected by 11.1% and 28.5%, respectively.

**Table 1:** Cross-tabulation of the numbers [n] of lesion types with percental distribution [%] at baseline (T0) and after 24 months of monitoring (T2) for the different tooth areas (mesiobuccal (mb), mesiolingual (ml), distobuccal (db), distolingual (dl) and if existing distal (d) cusp, as well as mesial (mr) and distal ridge (dr)

Area	Type	None (T2)	Cupping (T2)	Facet (T2)	Combined cupping-facet (T2)	Total (T0)
mb	None (T0)	2	0	4	0	6 (7.4%)
	Cupping (T0)	0	9	1	3	13 (16.0%)
	Facet (T0)	0	0	42	2	44 (54.3%)
	Combined cupping-facet (T0)	0	0	0	18	18 (22.2%)
	Total (T2)	2 (2.5%)	9 (11.1%)	47 (58%)	23 (28.4%)	81
ml	None (T0)	20	0	14	1	35 (43.2%)
	Cupping (T0)	0	2	1	0	3 (3.7%)
	Facet (T0)	0	0	41	0	41 (50.6%)
	Combined cupping-facet (T0)	0	0	0	2	2 (2.5%)
	Total (T2)	20 (24.7%)	2 (2.5%)	56 (69.1%)	3 (3.7%)	81
db	None (T0)	3	0	7	0	10 (12.3%)
	Cupping (T0)	0	0	0	0	0 (0%)
	Facet (T0)	0	0	62	2	64 (79.0%)
	Combined cupping-facet (T0)	0	0	0	7	7 (8.6%)
	Total (T2)	3 (3.7%)	0 (0%)	69 (85.2%)	9 (11.1%)	81
dl	None (T0)	15	0	8	0	23 (28.4%)
	Cupping (T0)	0	2	0	1	3 (3.7%)
	Facet (T0)	0	0	51	0	51 (63.0%)
	Combined cupping-facet (T0)	0	0	0	4	4 (4.9%)
	Total (T2)	15 (18.5%)	2 (2.5%)	59 (72.8%)	5 (6.2%)	81
d	None (T0)	6	0	1	1	8 (9.9%)
	Cupping (T0)	0	3	0	0	3 (3.7%)
	Facet (T0)	0	0	50	0	50 (61.7%)
	Combined cupping-facet (T0)	0	0	0	3	3 (3.7%)
	Total (T2)	6 (7.4%)	3 (3.7%)	51 (64.2%)	4 (4.9%)	64
mr	None (T0)	59	0	11	0	70 (86.4%)
	Cupping (T0)	0	0	0	0	0 (0%)
	Facet (T0)	0	0	11	0	11 (13.6%)
	Combined cupping-facet (T0)	0	0	0	0	0 (0%)
	Total (T2)	59 (72.8%)	0 (0%)	22 (27.2%)	0 (0%)	81
dr	None (T0)	54	0	9	0	63 (77.8%)
	Cupping (T0)	0	0	1	0	1 (1.2%)
	Facet (T0)	1	0	16	0	17 (21.0%)
	Combined cupping-facet (T0)	0	0	0	0	0 (0%)
	Total (T2)	55 (67.9%)	0 (0%)	26 (32.1%)	0 (0%)	81
Overall	None (T0)	145	1	68	1	215 (39.1%)
	Cupping (T0)	0	15	4	4	23 (4.2%)
	Facet (T0)	15	0	256	7	278 (50.5%)
	Combined cupping-facet (T0)	0	0	2	32	34 (6.2%)
	Total (T2)	160 (29.1%)	16 (2.9%)	330 (60.0%)	44 (8.0%)	550

Overall, an increase in macroscopically visible wear was observed, whereby areas without wear at T0 mainly developed facets. Areas that already had F at T0 mostly displayed them also at T2 and only rarely developed further into combined cupping-facet.

**Fig. 4:**
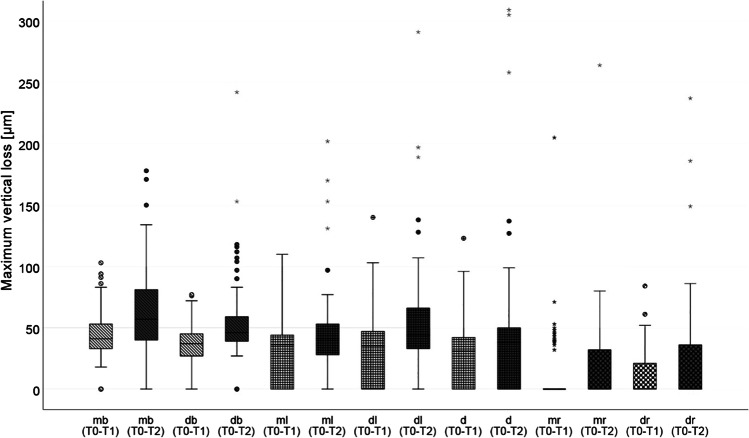
Boxplot diagram of the maximum vertical loss values [μm] for *n* = 81 study teeth after 12-month (T0–T1) and 24-month (T0–T2) observational period distributed to the seven areas (mesiobuccal (mb), distobuccal (db), mesiolingual (ml), distolingual (dl) and if existing distal (d) cusp as well as mesial (mr) and distal ridge (dr)). Buccal cusps are shaded oblique, lingual cusps are checkered small and ridges are checkered large; outliners (Ο), extreme values (*)

Loss values increased significantly at T2 compared to T1 for all areas (Fig. 4; for all areas *p*-values ≤ 0.001), although significantly less than in the first 12 months (T0–T1) of monitoring (mb, ml, db, dl and d *p* ≤ 0.001; dr *p* ≤ 0.05; mr n.s.). Loss values in the second observation period (T1–T2) were mb 15.0 (11.0; 18.0), ml 8.0 (0; 11.0), db 13.0 (8.0; 18.0), dl 10.0 (5.0; 16.0), d 3.0 (0; 10.0), mr and dr: 0 (0; 0). Loss values from T0–T1 did not correlate with those from T1–T2, except for d (*r* = 0.314: *p* ≤ 0.001) and dr (*r* = 0.366; *p* ≤ 0.001).

**Fig. 5:**
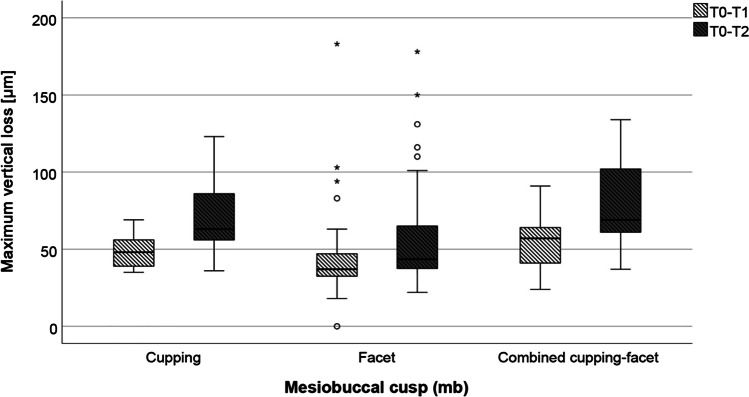
Loss values for the MB area from after 1 (light grey) and after 2 years (dark grey) depending on the wear morphology. Circles: outliers (up to one and a half times the box length range), star: extreme value (more than one and a half times the box length range)

When considering the mesiobuccal area, which showed the greatest variety of morphologies, significantly higher loss values were found for combined cupping-facet from T0 to T1 as well as from T1 to T2 than for those who had facets (*p* ≤ 0.001 and 0.05 respectively; Fig. 5).

The relation of loss values to possible associated aetiological factors is presented in Table 2. The only factor that had a significant association with the loss values was sex; males had significantly higher loss values than females and also had a higher proportion of C or CF compared to F (males: 51.5 % and 48.3% resp.; females: 32.7 % and 67.3% resp.); however, this did not reach significance (*p* = 0.104). All other factors were not significantly associated with loss values.

**Table 2:** Association between loss values (μm) from T0–T2 and T1–T2 (median and 95% confidence interval) and the various influencing factors that were surveyed with the questionnaires

Item		Category			*p* value
Sex	*n* (%)	Male 31 (38.3)	Female 50 (61.2)		
	Loss T0–T2 (μm) Loss T1–T2 (μm)	76.0 (60.0; 87.0) 24.0 (14.0; 30.0)	46.5 (42.0; 59.0) 12.5 (6.0; 16.0)		*p* = 0.020 *p* = 0.018
Wearing a night guard		No	Yes		
	*n* (%)	53 (65.4)	28 (34.6)		
	Loss T0–T2 (μm) Loss T1–T2 (μm)	59.0 (46.0;66.0) 14.0 (7.0;18.0)	48.0 (41.5;69.0) 16.0 (9.5;30.0)		*p* = 0.933 *p* = 0.585
Consumption of chewing gum		No	Yes		
	*n* (%)	29 (35.8)	52 (64.2)		
	Loss T0–T2 (μm) Loss T1–T2 (μm)	59.0 (41.0; 87.0) 11.0 (4.0; 28.0)	52.5 (45.0; 65.5) 16:0 (12.5; 19.0)		*p* = 0.961 *p* = 0.933
I like acidic food		No	Neither	Yes	
	*n* (%)	26 (32.5)	18 (22.5)	36 (45.0)	
	Loss T0–T2 (μm) Loss T1–T2 (μm)	60.5 (45.5; 72.0) 16.5 (13.0; 28.0)	68.0 (39.5; 95.9) 13.5 (1.5; 28.0)	50.5 (42.0; 64.0) 15.5 (7.0; 19.0)	*p* = 0.346 *p* = 0.639
I like acidic drinks	*n* (%)	42 (52.5)	19 (23.8)	19 (23.8)	
	Loss T0–T2 (μm) Loss T1–T2 (μm)	53.0 (44.0; 63.5) 15.0 (12.5; 19.0)	56.0 (36; 83.0) 16.0 (0.0; 28.0)	59.0 (42.0; 78.0) 16.0 (3.0; 24:0)	*p* = 0.906 *p* = 0.846

Also, when looking at the two groups (those who had cupping and combined cupping-facet compared to those who had no wear or facets), there was no significant difference in wearing a night guard (phi = 0.05; *p* = 0.654), consumption of chewing gum (phi = − 0.134; *p* = 0.228) or taste preferences (I like acidic food: phi = 0.140; *p* = 0.456; I like acidic drinks: phi = 0.112; *p* = 0.608).

No significant correlation between loss values of tooth wear after 12 and 24 months of monitoring and the daily acid impacts as calculated from the questionnaires at T0, T1 and T2. could be found (Table 3).

**Table 3:** Correlation between the loss values after 12 and 24 month of monitoring and the daily acid impacts as calculated from the questionnaires at T0, T1 and T2

		Acid impacts from all sources T0	Acid impacts from all sources T1	Acid impacts from all sources T2	Acid impacts from food T2	Acid impacts from drinks T2	Mean acid impacts from all sources (T0, T1, T2)
Loss T0–T1	Rho	0.007	0.053	0.066	0.015	0.109	0.019
	*p* value	0.954	0.636	0.560	0.897	0.334	0.869
Loss T0–T2	Rho	0.009	− 0.021	0.032	0.041	0.069	− 0.004
	*p* value	0.940	0.852	0.773	0.717	0.538	0.972
Loss T1–T2	Rho	− 0.056	− 0.094	− 0.044	0.029	− 0.030	− 0.057
	*p* value	0.625	0.405	0.695	0.800	0.790	0.612

## **Discussion**

The second publication of this observational study with data of 24-month monitoring showed a further progression of wear. Depending on the location of the cusps, loss values ranged between 38 μm (d) and 57 μm (mb) in the overall study period. This is less than data from an older study [8] that reported molar tissue loss values of 51 ± 48 μm after 12 months and of 91 ± 59 μm after 24 months. On the other hand, the data from the present study are higher than the wear rates reported in a material science context. A recent systematic review [26] on the wear of antagonistic enamel caused by different ceramic materials included 14 studies with patients aged between 18 and 73 years and observation periods between 3 and 36 months, looking at premolars and molars in the upper and lower jaw. In most of these studies, wear rates of antagonistic natural teeth were included as control showing an average wear of 9.6 μm per year. The loss values that have been monitored are within the range of published values, but overall, it is difficult to compare these data because there is no standardized method for measuring wear and as a result very different measurement methods and parameters are used.

The wear rate in the present study was not linear but decreased in the second year of follow-up. Similar was also shown in a longitudinal study on wear after the insertion of ceramic restorations [27]. In this study, plaster models were digitally imaged with a laboratory scanner 1, 2 and 3 years after the insertion of such restorations and superimposed on the baseline model. The wear of the restorations, their antagonists and an antagonistic contralateral tooth pair without restorations was determined. After 1 year, the wear of the latter was between 40 and 90 μm and decreased significantly in the next 2 years. These values correspond quite well to those found in the present study, although they seem to be somewhat higher overall. However, these were patients who had had a restoration placed, which could have influenced the wear rate. Furthermore, the volumetric behavior of the impression and model material as well as model imperfections such as small air inclusions or plaster beads may have had an influence.

A non-linear tissue loss was also found in the study mentioned above [8]. In this study, 20-year-olds were included and the maximum tissue loss in the area of antagonistic contact surfaces was monitored over 4 years. The tissue loss of the molars decreased from about 5 μm at the beginning to about 3 μm at the end of the study. The authors hypothesised that the force per area unit is reduced with increasing size of the wear area and that wear could be a self-limiting process. However, clearly more research is needed to improve the insight into the dynamics of wear.

As in the first year, the wear rate of combined cupping-facets was higher than that of other wear morphologies, so this defect shape may perhaps be considered a risk indicator for increased wear rates. For facets, lower rates of progression were found, but little is known about how facets proceed. Similar to wear in general, facets have mostly been investigated with clinical indices, while quantitative measurements have rarely been carried out. A Dutch study [7] compared plaster casts of young adults at 3-year intervals and measured the area of facets after imaging with a flatbed scanner. The mean increase in facet area ranged from 1.1 mm^2^ for anterior teeth and premolars to 3.4 mm^2^ for molars, with a considerable interindividual range. The increase in facet area was associated with a few orthodontic classifications for anterior teeth and premolars, but not for molars. Self-reported grinding or clenching did not have a significant effect on the increase in facet size for any group of teeth supporting the present finding that subjects with facets in the mesiobuccal area did not differ from those with cuppings or combined cupping-facets regarding wearing a night guard.

Cuppings are generally considered to be a sign of erosive tooth wear [28], but the relationship between acid exposure from nutrition and the prevalence of such lesions has not been demonstrated [29]. Longitudinal observational studies in particular have so far failed to show such an association between nutrition and lesion progression [15, 30, 31].

The present data could not show this association either, although different ways to characterize the nutrition behavior of study participants were used. Thus, instead of individual food- or beverage-related items combined overall acid impacts were analysed. Furthermore, not only the latest acid impacts at the end of the 2-year observation time but also the mean value from three survey points (T0, T1, T2) with the loss values were evaluated. In addition, participants were asked for taste preferences expecting a more comprehensive and representative picture of nutrition behavior overall compared to one-time surveys and queries of individual items. Nevertheless, the findings did not show a correlation between loss values and acid impacts as well as taste preferences.

One explanation could be that questionnaires are in general only partially suitable for recording nutrition behavior. For example, the analysis of data from the nutrition questionnaires from T0 and T1 [32] showed that there was little correlation between the data from the two time points, especially for the acid impacts. Taste preferences seemed to be a somewhat more stable measure, but, interestingly, there was no correlation between acid impacts and the corresponding taste preferences. On the one hand, these findings could indicate a problem that is inherent to questionnaires, among others the difficulty of remembering one’s behavior correctly or that answers can be influenced by social desirability [33]. One could therefore question whether self-reported nutrition behavior has anything at all to do with actual nutrition, and thus, memory-based survey methods in this context have generally been questioned [34].

On the other hand, nutrition habits may be highly variable, especially in younger adulthood [35], so there may be no continuous influence from nutrition at all.

What has to be considered as well is that cuppings may not be a characteristic sign or result of erosion. Wear morphologies associated with concavities are common in historical and prehistoric individuals [36] and the comparison of wear shapes in groups with different nutrition has shown that cuppings can occur in both subjects with abrasive and acid-rich nutrition [37] and thus may not be pathognomonic for erosive tooth wear.

Furthermore, it is not yet clear how such morphologies develop. Even though the observation period of 24 months is relatively short, the first indications that a facet evolves first before a cupping forms were shown. Until now, it was assumed that cuppings develop when dentin is exposed with increasing wear, which then wears out faster than the enamel due to its lower microhardness and thus forms a cupping [38]. At least, this theory was experimentally disproved because it was shown that such cuppings can also form within the enamel [39]. In this study, human molars were exposed to different loads at different pH values of the surrounding liquid in a chewing simulator. The formation of cuppings was clearly load and pH-dependent, but it was not investigated whether cuppings would also form in a neutral environment.

However, other factors that have not been in the focus so far may also play a role. For example, in a study of the macrowear patterns of pre-industrial and industrial burial remains, it was reconstructed that food properties may influence chewing behavior and that there is a biomechanical feedback loop between food texture, chewing behavior and wear [40]. Even though the differences in food texture in the periods studied were certainly much greater than would be expected in today’s usual range, asking about this aspect in nutrition questionnaires might reveal new links between nutrition and wear.

Another factor could be the bite force, which seems to be higher in males than in females [41] and which was also held responsible for the higher loss values in males. Bite force does not seem to play a role in the wear of antagonists of ceramics, but it does in the corresponding natural tooth pair [27].

There could also be a genetic disposition for wear [42–44]. Epidemiological data show relatively consistently that males are more frequently affected by erosive tooth wear than females [45] and this was also the case in the present study. Males not only had significantly higher loss values than females but also a higher percentage of cuppings or combined facts/cuppings (although the difference did not reach significance at this stage). The sex-dependent greater disposition to erosion could also be confirmed in an in vitro experiment [46] showing that tooth samples from male donors had higher loss values with the same acid exposure. Furthermore, gene analyses show significant correlations between variations in enamel-forming genes and the lower susceptibility to erosion in female subjects. Amelogenin, for example, is an enamel-forming protein encoded by sex chromosomes x and y. This link is supported from another direction, as it has been shown that it is possible to determine sex from sex chromosome-linked isoforms of amelogenin [8]. However, beyond sex, genetic factors could certainly influence wear in a variety of ways. However, a considerable body of research is needed in this field before such assumptions can be regarded as reliable.

Taken together, all this suggests that tooth wear appears to be an extremely complex process in which many more factors may play a role than have been investigated so far. It might therefore be worthwhile to rethink both the theoretical constructs of tooth wear as well as to improve the research methods for investigating wear and its triggering factors.

Even though the association between tooth wear and the assumed aetiological factors is not entirely clear, a few principles can be derived that could be helpful for the prevention of tooth wear [47]. Since any form of wear will cease if the triggering noxious agent is identified and avoided as best as possible, the focus is on causal intervention approaches. In the case of erosive tooth wear, this could involve reducing acid exposure, for example through dietary changes or treatment of reflux disease. In addition, fluorides in combination with stannous ions can reduce the acid solubility of dental hard tissues and thus prevent erosion [48]. If attrition or abrasion is the main factor, efforts should be made to avoid the triggering mechanical action. However, all these measures are only indicated if the wear rate is pathological [47], so monitoring wear with intraoral scanners can certainly be a diagnostic tool in this context. However, this would also require more studies that collect data over longer periods of time so that clinically relevant concepts for wear patterns can be developed. In addition, monitoring wear with intraoral scanners would also be important in answering the question of whether and which interventions are successful in reducing wear. If restorative interventions are considered, they should be implemented as late and as minimally invasive as possible [49].

As a limitation of the present study setup, the sole analysis of the occlusal surface of a single mandibular first molar has to be discussed. The authors are aware that tooth wear can affect all teeth in the dental arch and different surfaces. However, at the beginning of the study in 2018 transfer inaccuracies of full-arch impressions for IOS have been described and are even today challenging [50, 51]. Furthermore, multicenter studies are required to monitor a larger group of participants to gain more information about the development and process of tooth wear.

## **Conclusion**

Observation data of tooth wear show a further progression after 24 months, which was, however, less than in the previous 12 months. Males had significantly higher wear rates than females, but neither nutrition habits nor the wearing of a night guard nor the consumption of chewing gum were related to loss values. Morphologically, there were mainly facets and only a small number of cuppings or combined facets/cuppings, which were located almost exclusively on the mesiobuccal cusp. The latter presented the highest loss values and could thus be a risk indicator for higher wear rates.

## Data Availability

Data are available from the corresponding author on reasonable request.
